# Grit protects medical students from burnout: a longitudinal study

**DOI:** 10.1186/s12909-020-02187-1

**Published:** 2020-08-12

**Authors:** Muhammad Raihan Jumat, Pierce Kah-Hoe Chow, John Carson Allen, Siang Hui Lai, Nian-Chih Hwang, Jabed Iqbal, May Un Sam Mok, Attilio Rapisarda, John Matthew Velkey, Deborah Lynn Engle, Scott Compton

**Affiliations:** 1 Office of Education, Duke-NUS Medical School, 8 College Rd., Level 3, Singapore, 169857 Singapore; 2 Singapore General Hospital, Singapore, Outram Road, Singapore, 169608 Singapore; 3 Department of Psychology and Neuroscience, Duke University School of Medicine, North Carolina, 27708 Durham USA

**Keywords:** Grit, Burnout, Medical education, Tolerance for ambiguity, Engagement, Medical school

## Abstract

**Background:**

Burnout is a serious issue plaguing the medical profession with potential negative consequences on patient care. Burnout symptoms are observed as early as medical school. Based on a Job Demands-Resources model, this study aims to assess associations between specific job resources measured at the beginning of the first year of medical school with burnout symptoms occurring later in the first year.

**Methods:**

The specific job resources of grit, tolerance for ambiguity, social support and gender were measured in Duke-NUS Medical School students at the start of Year 1. Students were then surveyed for burnout symptoms at approximately quarterly intervals throughout the year. Using high ratings of cynicism and exhaustion as the definition of burnout, we investigated the associations of the occurrence of burnout with student job resources using multivariable logistic regression analysis.

**Results:**

Out of 59 students, 19 (32.2%) indicated evidence of burnout at some point across the first year of medical school. Stepwise multivariable logistic regression analysis identified grit as having a significant protective effect against experiencing burnout (Odds Ratio, 0.84; 95%CI 0.74 to 0.96). Using grit as a single predictor of burnout, area under the ROC curve was 0.76 (95%CI: 0.62 to 0.89).

**Conclusions:**

Grit was identified as a protective factor against later burnout, suggesting that less gritty students are more susceptible to burnout. The results indicate that grit is a robust character trait which can prognosticate burnout in medical students. These students would potentially benefit from enhanced efforts to develop grit as a personal job resource.

## Background

Since May 2019, the World Health Organization (WHO) has included burnout in the 11th Revision of the International Classification of Diseases (ICD-11) as an occupational phenomenon. Burnout is conceptualized and defined as an unsuccessfully managed syndrome resulting from chronic workplace stress [1]. Burnout is typically characterized by emotional exhaustion, cynicism and/or a perception of reduced professional accomplishment [1, 2]. Individuals in careers requiring close interactions with other people are most susceptible to burnout [3], this makes healthcare workers particularly prime for developing burnout [4].

Numerous reports have shown that medical professionals all over the world at all career stages are succumbing to the scourge of burnout [4–10]. Doctors experiencing burnout fall ill more frequently, are more prone to making medical errors, and have a higher tendency to leave the profession [11]. In extreme conditions, burnout can result in depression which adversely affects physician health and performance [12]. When physician health deteriorates, patient care suffers [13].

Burnout can be observed as early as medical school, where medical students from various countries are reporting higher incidences of burnout when compared to their non-medical student counterparts [6]. Students experiencing burnout have shown inability to connect with others [14], a strong desire to leave the institution and the profession [15, 16], and higher likelihood of suicidal ideation [17]. This observation is particularly distressing considering that matriculating medical students are generally optimistic and engaged [18]. Following matriculation, medical students report increasing incidence of burnout throughout the course of medical school [19, 20]. Reasons for the high burnout rate amongst medical students include the heavy cognitive load, intense competition among classmates, and constant exposure to death and suffering [21, 22].

While the symptoms and effects of burnout have been previously established, factors which may help mitigate or predict burnout are less obvious. One recently suggested potential mitigating factor for burnout is grit, defined as passion and sustained persistence applied toward long-term achievement [23]. However, the evidence suggesting this association was from a cross-sectional study of doctors in the United Kingdom [24]. Nonetheless, the potential for grit—a modifiable factor [25], represents an enticing target to mitigate burnout. Other factors which have been shown to affect psychological well-being and improved life satisfaction include tolerance for ambiguity (TFA) [26], religiosity [27, 28], and social support [29].

The primary aim of this study is to determine if grit, tolerance for ambiguity (TFA), religiosity and/or social support are predictive of burnout in year one medical students. The secondary aim is to determine the prognostic utility of these factors in predicting burnout in the first year of medical school. We hypothesize that a student possessing protective resources will be at lower risk of reporting incidences of burnout over the course of an academic year. Students lacking protective job resources would be at a higher risk of burnout and thus should be monitored regularly. In assessing risk of burnout, we carried out a panel survey of Year 1 students entering Duke-NUS Medical School and typed them for the character traits listed previously at the start of the academic year. These students were subsequently followed up for incidences of burnout at three time points during the academic year.

## Methods

### Conceptual framework

The conceptual framework of the study is based on the Job Demands-Resources model [30]. In this model every job has its own specific risk factors which are classified as job demands and job resources.

Job demands are psychological, physical, social or organizational aspects of the job which require sustained physical and/or psychological effort or skills and are therefore associated with certain physiological and/or psychological costs.

Job resources refer to physical, psychological, social or organizational aspects of the job that function in achieving work goals, reduce job demands and the associated psychological and physical costs and/or stimulate personal growth and development. These resources may be located at the level of the organization (e.g., salary, career opportunities, job security), interpersonal and social relations (work support groups, work team dynamics), work organization (role clarity), or the level of the task (autonomy, performance feedback).

In general, job resources and job demands are antagonistic. Job demands, such as high work pressure and emotionally demanding interactions, would necessitate mobilisation of job resources. Recent publications have shown that personal resources as well have an antagonistic role to job demands and hence should be categorized as job resources [31–33]. An individual possessing sufficient resources to support the demands of a job is engaged and not emotionally strained. But when resources are lacking, the individual is at risk of developing emotional exhaustion, cynicism, and reduced work efficacy which are a prelude to burnout [30, 34]. This is further exacerbated by poor working conditions, a high work load combined by understaffing and a perceived lack of control as often experienced by healthcare workers and medical students.

Resources measured in this study, which can be invoked by medical students to parry the intense demands of medical school, are grit, religiosity, social support and comfort with uncertainty. While we recognise that the choice of resources is not exhaustive, these factors have been shown to be protective against burnout in the literature. Grit is defined as passion and sustained persistence applied toward long-term achievement [23, 35]. The relationship between grit and burnout has been expounded in the background section. Religiosity is defined as the adherence to beliefs, doctrines, ethics, rituals, texts, traditions, and practices related to a higher power and associated with an organized group [36]. Studies on religiosity and burnout amongst medical students have shown conflicting results with some indicating lack of a relationship [37] and others showing an inverse relationship [38, 39]. Social support is the perception of the quality of emotional support provided by others [40]. Studies have also indicated that medical students who perceive a lack of social support have a higher tendency to undergo depression and burnout [41]. TFA is defined as the degree to which an individual is comfortable operating under conditions of uncertainty, unpredictability, conflicting directions and multiple demands [42]. Physicians with low TFA scores are more prone to develop burnout [43], but this correlation has not been reported in medical students. Lastly, burnout rates were compared to the gender make-up of the class. Studies on gender differences and mental health in medical students have shown varying results. Some studies have shown that no difference exists between the sexes in predicting burnout [44, 45]. Some report that males are at a higher risk [20, 46], while some indicate that female medical students are more vulnerable [47, 48].

### Methodology /student survey

A study of the class of 2021 students at Duke-NUS Medical School during year 1 was conducted using survey methodology. Duke-NUS Medical School is an American style, graduate-entry, allopathic medical school in Singapore. The panel survey consisted of demographic questions with questionnaires measuring the four job resources of grit, religiosity, social support and TFA, as well as symptoms of burnout.

Burnout was measured using the validated Maslach Burnout Inventory-Student Survey (MBI-SS), which has become the gold-standard for defining burnout, especially amongst medical students [17, 49–51]. The MBI-SS uses three different subscales: Emotional Exhaustion, Cynicism and Personal Inefficacy with a high score on any of these sub-scales indicating burnout [2]. Grit was measured by a 12-item Grit scale [35]. An uncorrected score of 60 indicates that an individual is extremely gritty while the lowest score on the scale indicates that the individual is not gritty at all [23]. Similar to other studies [52, 53], grit is expressed as a single factor. Religiosity was measured using the Duke University Religion Index (DUREL), which is a five-item scale made up of three dimensions: intrinsic religiosity (3 items), organizational religiosity (1 item), and non-organizational religiosity (1 item), measured on a 5-point Likert scale [54]. The Multidimensional Scale of Perceived Social Support (MPSS) was used to measure the availability of social support an individual perceives they are receiving from the family, friends and a significant other [55]. TFA was measured by a seven-item TFA scale (TAS). The TAS measures the student’s ability to cope with situations of uncertainty. Students ranked their responses to the 7 items on the TAS on a 6 response Likert scale. Scores ranged from 7 (lowest tolerance for ambiguity) to 42 (highest tolerance for ambiguity) [56].

The survey was created on an online platform, and a link to the survey was sent out to the Duke-NUS medical school mailing list. The first sampling point (T_1_) was from 22nd August – 5th September 2017, at which time all 4 job resources and burnout were surveyed. For the next three sampling points, 1st November – 10th November 2017 (T_2_), 1st February – 10th February 2018 (T_3_), and 1st May – 10th May 2018 (T_4_), only burnout was surveyed. Students indicated their consent electronically before attempting the survey. The study was approved by the National University of Singapore Institutional Review Board.

### Statistical analysis

Baseline characteristics were summarized using the mean and standard deviation (SD) for burnout and the job resources (grit, religiosity, social support and TFA). To assess the internal consistency of responses, Cronbach’s alpha was calculated for each scale (or subscale in the case of burnout). Summary statistics of survey instruments are reported for all responders in terms of numbers and percentages or mean scores and standard deviations, as appropriate.

Univariate logistic regression analysis was conducted on grit, religiosity, social support, and TFA to assess protective associations against burnout. For the purpose of this study, a student was considered as experiencing burnout if they scored high on both the emotional exhaustion and cynicism sub-scales of the MBI-SS, as used by several recent studies [24, 57, 58] . A student that did not register high on both of these sub-scales was scored as ‘NO BURNOUT’. Conversely, students scoring high on any of these sub-scales at least once in the study period are labelled as ‘BURNOUT’. While burnout is not recognised as a distinct disorder in the Diagnostic and Statistical Manual of Mental Disorders (DSM) [59], it is recognised in the 11th revision of the International Classification of Diseases (ICD-11) [1]. This allowed for the tentative classification of BURNOUT or NO BURNOUT according to the responses to the MBI-SS. To address the first research objective, the resources of grit, religiosity, social support and TFA were compared between students who did not experience burnout to those who reported burnout at least once during the year. Results were summarized as odds ratios (ORs) and 95% confidence intervals (CIs) reflecting the protective effect for NO BURNOUT. Predictive capabilities of resources demonstrating statistically significant protective effects against burnout (NO BURNOUT) were summarized using a Receiving Operating Characteristic (ROC) curve and negative (NPV) and positive predictive value (PPV) with a statistically optimal cut-point indicated by the Youden J-statistic. All statistical analyses were performed with SAS version 9.4. Statistical significance was set at *P* < 0.05.

## Results

### Reliability statistics

Internal reliability of survey instruments used in this study was compared to previous values found in the literature. The obtained reliability statistics (Cronbach’s alpha) for the MBI-SS scale were 0.76, 0.85, and 0.76 for emotional exhaustion, professional efficacy and cynicism, respectively. This is similar to previously reported Cronbach’s alpha: 0.76, 0.90, and 0.76 [60]. The Cronbach’s alpha received from the TFA scale was 0.81, while other studies have previously reported 0.75 [42]. The responses from the DUREL scale in this study had a Cronbach’s alpha of 0.90, previous studies have a range of 0.78 to 0.91 [61]. The Cronbach’s alpha from the Grit scale used in this study was 0.81, while other studies have showed a range of 0.77 to 0.85 [23]. The four different sub-scales of the MPSS generated Cronbach alpha values of 0.95, 0.84, 0.91, and 0.87 for significant other, family friends, and overall, respectively. Previous studies have shown a value range of 0.88 to 0.92 [62]. Overall, the obtained reliability statistics (Cronbach’s alpha) closely matched previously reported values.

### Descriptive statistics

The panel survey attained a response rate of 93.7% (59/63 students) for all timepoints. Fifty-nine out of 63 students (response rate 93.7%) responded to the surveys at all timepoints. None of the respondents dropped out of the study. The responses of these 59 students were analysed in this study. Descriptive statistics for survey instruments are shown in (Table 1). Burnout, religiosity, and social support are listed by their respective subscales. When only considering students who reported high scores on the emotional exhaustion and cynicism subscales, 19 students reported high MBI-SS scores at least once over the course of the academic year. This constitutes 32.2% out of the 59 responders.

**Table 1:** Summary of survey instrument results (mean ± standard deviation) at the 4 study sampling times (*N* = 59)

Survey Instrument	T_**1**_	T_**2**_	T_**3**_	T_**4**_
**BURNOUT**				
Emotional Exhaustion	13.2 ± 5.2	13.2 ± 4.9	11.6 ± 5.4	11.8 ± 4.6
Professional Efficacy	25.6 ± 5.9	23.8 ± 6.0	24.8 ± 4.9	24.8 ± 5.5
Cynicism	6.4 ± 4.7	7.7 ± 5.7	8.1 ± 6.1	7.6 ± 5.6
**TOLERANCE FOR AMBIGUITY**	20.6 ± 5.5	–	–	–
**RELIGIOSITY**				
Organizational Religious Activity (ORA)	2.8 ± 1.8	–	–	–
Non-Organizational Religious Activity (NORA)	2.5 ± 1.8	–	–	–
Intrinsic Activity (IR)	8.6 ± 4.0	–	–	–
**GRIT**	3.6 ± 0.5	–	–	–
**SOCIAL SUPPORT**				
Significant Other	5.6 ± 1.3	–	–	–
Family	5.7 ± 1.0	–	–	–
Friends	5.7 ± 0.8	–	–	–
Overall	5.7 ± 0.8	–	–	–

T_1_: 22nd August – 5th September 2017, T_2_: 1st November – 10th November 2017, T_3_: 1st February 2018- 10th February 2018, T_4_: 1st May – 10th May 2018

### Multivariable statistical analysis

Subsequent analysis compared demographics between the different job resources, as well as gender, for the predicted outcome of ‘NO BURNOUT’. The statistical analyses focused on the 40 students who had low MBI-SS scores throughout the study period versus the 19 students who reported at least 1 incidence of burnout over the academic year. Stepwise multivariable logistic regression analysis identified grit as the single job resource significantly predictive of the NO BURNOUT outcome (*p* < 0.01). A one-unit increase in grit was reflected in a 19% average increase in the odds of NO BURNOUT (OR = 1.19; 95% CI: 1.04, 1.36) (Table 2).

**Table 2:** Analysis of study variables as predictors of NO BURNOUT

Parameter	CHI SQUARE		ODDS RATIO ESTIMATES			
	Wald Chi-Square	***p***-value	B-Value	Point Estimate	95% Wald Confidence Limits	
**Female Gender**	0.48	0.49	0.45	1.56	0.44	0.45
**Tolerance for Ambiguity**	0.06	0.81	0.02	1.02	0.90	0.02
**Religiosity**	0.31	0.58	−0.05	0.95	0.81	−0.05
**Grit**	6.77	< 0.01	0.17	1.19	1.04	0.17
**Social Support**	0.77	0.38	0.03	1.03	0.96	0.03

**Fig. 1:**
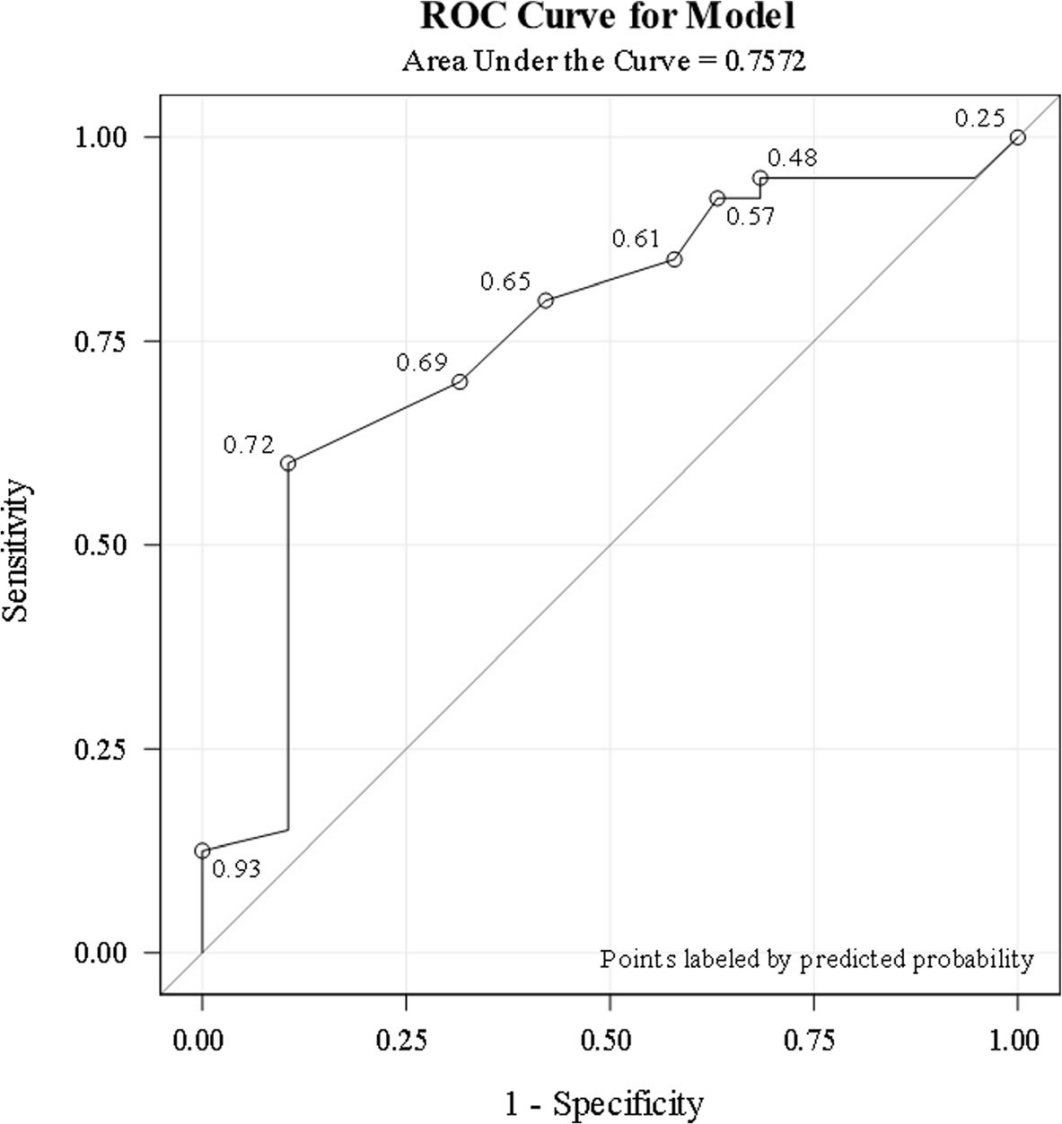
Receiver Operating Characteristic (ROC) Curve for grit score as a predictor of NO BURNOUT’. Area under curve (AUC; (95% Confidence Interval): 0.76 (0.63, 0.89))

**Fig. 2:**
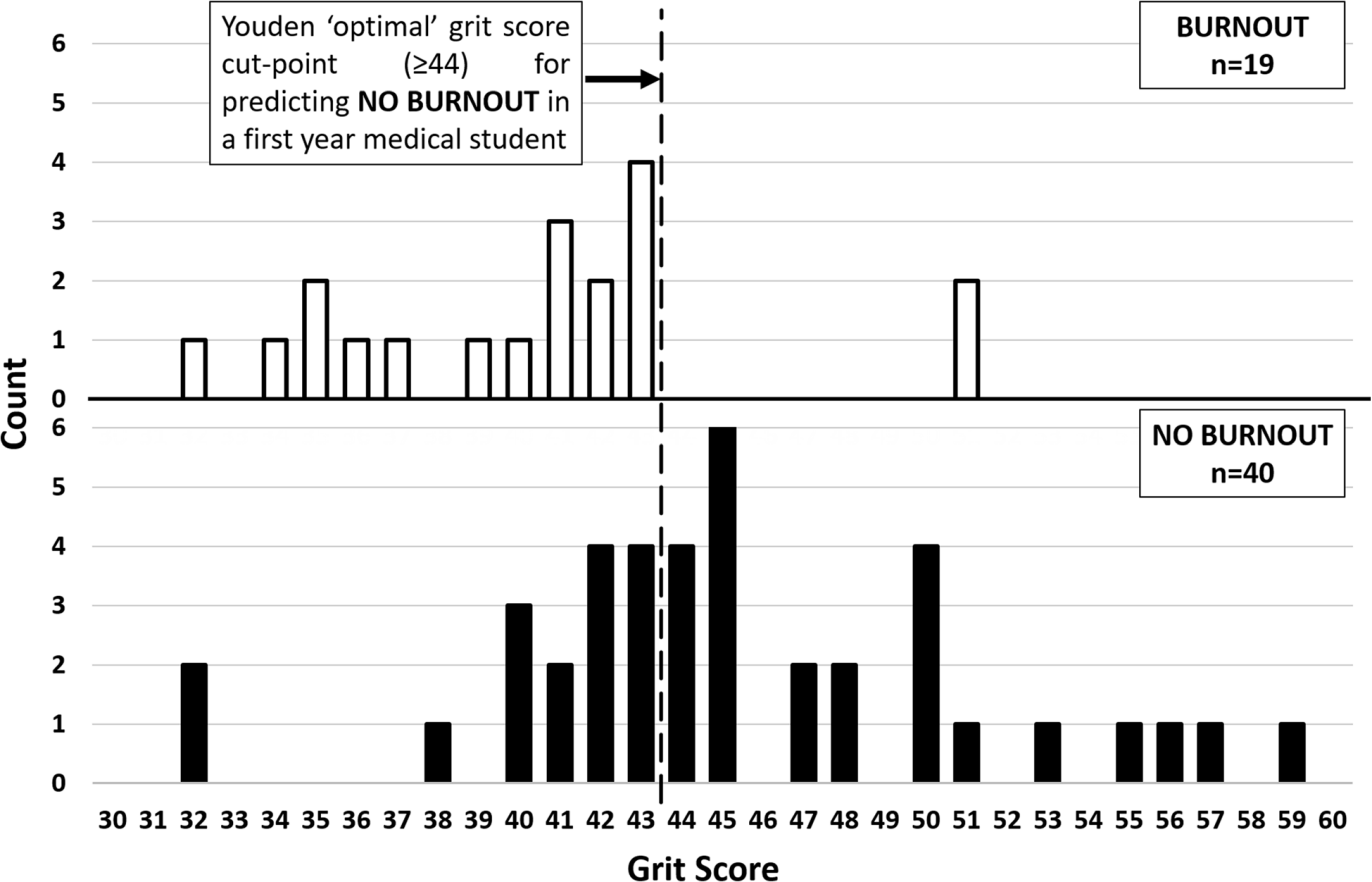
**D**istribution of students according to their uncorrected grit score (x-axis). Y axis shows student count. Upper panel: students experiencing burnout at least once in the year: lower panel: students experiencing NO BURNOUT

Area under the grit ROC curve as a predictor for NO BURNOUT was 0.76, (95% CI: 0.63, 0.89) (Fig. 1). An uncorrected grit cut-off score of 44 was indicted as the statistically optimal threshold for discrimination of NO BURNOUT (Youden J-statistic = 0.49), with associated positive and negative predictive values for NO BURNOUT outcome of PPV = 0.92 and NPV = 0.52, respectively (Table 3). Only 2 of 26 students (7.7%) with grit score ≥ 44 reported high MBI-SS scores. Conversely, 33 students had grit score < 44, with 17 (51.5%) reporting high MBI-SS scores and 16 (48.5) had low MBI-SS scores (Fig. 2).

**Table 3:** ROC Curve cut-points with positive and negative predictive values (NPV and PPV) for predicted outcome of NO BURNOUT

GRIT Score*	PPV	NPV	Youden J
32	0.68	.	0.00
34	0.68	0.33	0.00
35	0.70	0.60	0.11
36	0.72	0.67	0.16
37	0.73	0.71	0.21
38	0.75	0.75	0.27
39	0.74	0.67	0.24
40	0.76	0.70	0.29
41	0.76	0.57	0.27
42	0.80	0.58	0.38
43	0.82	0.52	0.38
**44**	**0.92**	**0.52**	**0.49**
45	0.91	0.46	0.39
47	0.88	0.40	0.24
48	0.86	0.38	0.19
50	0.83	0.36	0.14
51	0.75	0.33	0.04
55	1.00	0.35	0.10
56	1.00	0.34	0.08
57	1.00	0.33	0.05
59	1.00	0.33	0.03

*Uncorrected grit score values (Grit Score × 12)

An uncorrected grit cut-off score of 44 was indicated as the statistically optimal threshold for discrimination of NO BURNOUT (in bold)

## Discussion

Grit was the only job resource studied that exhibited a correlation with a reduced risk of burnout. That is, a grittier student is less likely to score high on the MBI-SS over the course of the first academic year of medical school. The AUC of 0.76 under the ROC curve indicates that grit would be considered a moderately accurate predictor of burnout among medical students. Interestingly, grit was only measured at the start of the school year, and yet it allowed for the accurate prediction of the scores on the MBI-SS, across four different timepoints across the year. School administrators might use the grit scale to predict and anticipate for burnout amongst medical students.

Regarding the second research question, based on study results, a student having a grit score of ≥44 would have an estimated 92% chance of not experiencing burnout. Students having a grit score of < 44 are indicated to have almost equal probability of experiencing burnout or not. Hence high grit, as a predictor, has high specificity and low sensitivity in predicting NO BURNOUT. The data from this study points to high grit as an effective job resource in preventing the onset of burnout in medical students.

We have chosen to identify students with high scores of emotional exhaustion and cynicism as having burnout. Personal inefficiency, the third component of burnout, was not considered as emotional exhaustion and cynicism have been shown to be more strongly linked to burnout [63–67]. Several other studies have also chosen to only use emotional exhaustion and cynicism as indicators of burnout [24, 57, 58]. The conclusion that grit and burnout are correlated is also reached when comparing cynicism scores only. This indicates that cynicism is an underlying construct that is important in the relationship between grit and burnout.

Factors such as gender, religiosity, and social support have been shown to associated with burnout (or the lack thereof) [26–29]. Similar to the majority of studies dedicated to gender, the results of this study show that gender shares no relationship with burnout [20, 46, 68–70].

While TFA, social support and religiosity have shown to be protective against burnout in several studies [26–29], our results indicate no correlation. This could be an artefact due to the small sample size of our study. There could exist other nuanced factors which might interact with these resources which have not been investigated.

Our study is aligned with other studies which suggest that grit is a protective factor against burnout. Previous studies have shown that gritty doctors in the UK display less burnout [24]. Surgical residents in the USA that have a below-median level of grit tend to report not being satisfied with their residency and also have a higher attrition rate [71]. A similar study in Australia proved that GP registrars with high levels of resilience are associated with less burnout, anxiety, and stress [72]. These studies were cross-sectional and did not consider the temporal effect of grit on burnout. This current study, shows that grit is able to predict the MBI-SS scores of medical students over an academic year, further fortifying the link between grit and burnout.

To the best of our knowledge, this study is the first to demonstrate a relationship between grit and burnout in a Singaporean medical school. Singapore is a highly-stressed country [73–75], with a good percentage of healthcare workers admitting to being ‘burnt-out’ [76–79]. The job demands in medical school and the healthcare sector have been proven to be overwhelming and result in burnout if not managed appropriately. Hence, identifying any protective factors against burnout in such a highly-strung society is key for the mental well-being of Singaporean healthcare workers. Training to improve one’s grit might assist in ameliorating the immense job demands and hence preventing the onset of burnout.

Short term solutions to burnout, such as getting time off from work, may not necessarily solve the problem of burnout [80]. In a recent study, burnout rates did not differ when French urologists were given ‘protected time’ away from work [81]. This would indicate that burnout is not a result of overworking. Efforts to improve and develop grit might provide a better chance to protect against burnout than short-term, temporary solutions. More attention should be channeled to developing grit amongst medical students.

Individuals working towards a common goal, given ample opportunity for deliberate practice and reminders of their shared purpose have been suggested to develop grit as a group [82, 83]. The formation of a culture which promotes and breeds grit within an organization would be a stronger force to withstand burnout rather than just training individuals to be grittier. The setting up of a positive grit culture in medical school might reduce the problem of burnout. Grittier students fare better in exams, find a sense of purpose in their desired vocation, and are more optimistic than less grittier students [82, 84]. All of these characteristics are traits which one expects from a competent physician.

## Limitations affecting conclusions

This longitudinal study has allowed us to reach 2 main conclusions; 1) grittier students have a lower chance of facing burnout, and 2) a medical student with a grit score of ≥44 has a 92% chance of not developing burnout. However, these conclusions were made through the lens of a single class with a small sample size (*n* = 59) in a single institution. The small sample size was further exacerbated by the lack of 100% response rate, which is common shortfall in online data collections [85]. The missing 6.3% could have skewed the results differently. Other disadvantages of online collections include a short attention span and the inability to confirm the identiy of the survey taker. Burnout sampling points were also roughly aligned with the ends of courses. Burnout incidences might have differed if sampling points were changed. As a quantitative survey, we were limited in our ability to explore the rationales behind the student responses which could be more complex than the resources which were incorporated in the conceptual framework. A larger class size, and a study over multiple years, and replicating this study at multiple institutions would be required to validate the conclusions reached in this study as applicable to medical students in general.

## Data Availability

The datasets used and/or analysed during this study are available from the corresponding author upon reasonable request.

## Abbreviations

DUREL: Duke University Religion Index
MBI-SS: Maslach Burnout Inventory – Student Survey
NPV: Negative Predictive Value
NUS: National University of Singapore
PPV: Positive Predictive Value
ROC: Receiving Operating Characteristic
TFA: Tolerance For Ambiguity
TBL: Team-Based-Learning
TAS: Tolerance for Ambiguity Scale
